# Editorial for the Special Issue on Nanofabrication with Focused Electron/Ion Beam Induced Processing

**DOI:** 10.3390/mi12080893

**Published:** 2021-07-28

**Authors:** Rosa Córdoba

**Affiliations:** Institute of Molecular Science (ICMol), University of Valencia, 46980 Paterna, Spain; rosa.cordoba@uv.es

Focused electron beam (FEB) and focused ion beam (FIB) technologies have opened novel paths for material science research and technology at the micro and nano scales in recent decades. These technologies are highly adaptable, and flexible tools are used successfully in fundamental and applied research projects allowing visualization, elemental and structural analysis, and additive and subtractive manufacturing, as well as efficient modification of materials. All of this can be done on scales ranging from hundreds of μm to one nanometer. The unique flexibility of focused beams enables them to be used for rapid prototyping as well as nanostructure editing and analysis. Today, these technologies play an important role in the development of new integrated circuits based on miniaturized electronic devices.

Concerning the additive nanofabrication, the use of gas injection systems with advanced programs (total control over the beam scanning parameters) allow to grow in single-step nanostructures in three dimensions by the partial decomposition of a precursor material produced by the effect of the beam scanning. This technique is called focused ion/electron beam-induced deposition (FIBID/FEBID). Nanostructures grown using this additive manufacturing technique can have arbitrary shapes, both in-plane (down to 10 nm in lateral resolution) and out-of-plane (down to 30 nm in diameter and an aspect ratio, length/diameter, up to 200), and also can host insulating, metallic, ferromagnetic, or superconducting properties.

Accordingly, this Special Issue showcases seven research articles and one review article that focus on the current status of nanofabrication using focused electron/ion beam induced processing. These articles particularly explore the following aspects of these technologies: (1) purification of FEBID nanostructures [1], (2) high-resolution nanostructures using FEBID [2], (3) a new approach for the nanofabrication of metallic nanostructures based on FEB [3], (4) tuning of diameters in 3D FEBID nanostructures [4], (5) evolution of the microstructure and resistivity in Pt Ga^+^ FIBID deposits with in situ heating [5], (6) nanofabrication using Ga^+^ FIB-induced processing for photonic applications [6], and (7) fabrication of 3D FIBID nanostructures using He^+^ FIB [7,8].

(1) Purification of FEBID nanostructures: Markus Rohdenburg et al. [1] have reported a systematic study for the partial removal of hydrocarbon contaminations of deposits fabricated by FEBID via the formation of a carbon nitride matrix.

(2) High-resolution nanostructures using FEBID: Sangeetha Hari et al. [2] have developed a successful strategy to obtain high-resolution dense lines patterned by FEBID. To do so, authors have used focused electron beam-induced etching for the removal of the inconvenient interconnecting material formed between the lines as a result of the Gaussian profile of the beam.

(3) A new approach for the nanofabrication of metallic nanostructures based on FEB: Luisa Berger et al. [3] have proposed direct electron beam lithography to grow metallic nanostructures. This approach is based on the condensation of a low-volatile precursor based on copper onto the substrate at room temperature. After FEB irradiation, the non-irradiated resist is removed by sublimation or rising. The growth rate of deposits using this approach is higher than FEBID, and the composition is similar.

(4) Tuning of diameters in 3D FEBID nanostructures: Lukas Seewald et al. [4] have optimized a method for tuning diameters of individual 3D nanowires. In particular, authors have introduced beam blurring for controlled upward scaling and study the behavior at different inclination angles.

(5) Evolution of the microstructure and resistivity in Pt FIBID deposits with in situ heating: Chaorong Zhong et al. [5] have reported an in situ combined study of the microstructure and resistivity of the Pt FIBID deposits as a function of heating. Authors have found the annealing effect is recommended for the fabrication of metallic interconnectors.

(6) Nanofabrication using Ga^+^ FIB-induced processing for photonic applications: Mariachiara Manoccio et al. [6] have written a complete review of the nanofabrication of chiral-shaped structures using FIB induced processing for photonic applications. Authors have described the current fabrication strategies in order to tailor the dimensions of nanostructures and thus fine-tune their chiro-optical properties over a broad spectral region.

(7) Fabrication of 3D FIBID nanostructures using He^+^ FIB: Alex Belianinov et al. [7] have reported a detailed study of the effect produced on the resultant segment and angle growth for 3D nanoobjects varying according to several deposition parameters, such as beam current, dwell time, and pixel spacing. Authors have found heating effects that impact the segment angle and the feature size of complex 3D structures. On the other hand, Frances Allen et al. [8] have reported the fabrication and characterization of 3D insulator nanostructures with a high aspect ratio. Particularly, she has grown nanopillars and nanocylinders that exhibit charge induced branching, and moreover, she has analyzed their microstructure by transmission electron microscopy.

I would like to thank all authors who submitted their papers to this Special Issue. I would also like to thank all the reviewers for dedicating their time to provide careful and timely reviews to ensure the quality of this Special Issue.
